# Burden of Hypertension in the Capital of Afghanistan: A Cross-Sectional Study in Kabul City, 2015

**DOI:** 10.1155/2017/3483872

**Published:** 2017-01-03

**Authors:** Khwaja Mir Islam Saeed

**Affiliations:** Grant and Service Contract Management Unit (GCMU), Ministry of Public Health, Kabul, Afghanistan

## Abstract

*Background.* This study had the objective to assess the prevalence and associated factors of hypertension in an urban setting, Kabul city, Afghanistan.* Materials and Methods.* The World Health Organization's STEP-wise approach was adopted and used in Kabul in November 2015. The study analyzed a sample of 1172 adults in the age group of 25–70 years. Demographic, socioeconomic, and behavior data were collected using a structured questionnaire. Fasting venous blood sample was collected to assess the lipid profile and fasting blood sugar.* Results.* The study showed that the prevalence of hypertension among adult Kabul citizens was 32.3%. From this figure, 599 (51.1%) were females and 573 (48.9%) males with a mean age of 38.6 ± 12.2 years. Illiteracy rate was 49.6% and 77.5% were married. Smoking in adults were 8.1% and mouth snuff users were 9.8%. More than half (57.6%) of the study respondents were overweight and obese and 9.1% were recorded having raised blood sugar. In the multivariate logistic regression analysis, age, general obesity, central obesity, smoking, moderate physical activity, and taking fruits 3 days or less weekly were statistically significant predictors of hypertension.* Conclusions.* Burden of hypertension is increasing in main urban settings in Afghanistan. Integrated intervention focusing in main modifiable risk factors is needed to detect and prevent hypertension.

## 1. Introduction

Noncommunicable diseases (NCDs) are a major cause of morbidity and mortality globally. In contrary to the common misconception, the burden of these diseases is the worst in low- and middle-income countries where 80% of all NCDs occur, and it is rising rapidly [1, 2]. Hypertension (HTN) is one of those NCDs which is an important public health problem in both economically developed and developing nations [3]. As per World Health Organization report, about 40% of people aged more than 25 years had hypertension in 2008 [4]. Worldwide, 7.6 million premature deaths were attributed to high blood pressure [5]. It has been a main health challenge due to high frequency and associated risks for cardiovascular and kidney diseases such as myocardial infarctions, strokes, and renal failures [6] and even at the top end of the normal range increases the risk and is called silent killer [7, 8]. Predictors of hypertension include family history, age, race, obesity, and physical inactivity, lack of exercise, cigarette smoking, excessive salt intake, and excessive alcohol intake [9, 10]. Some of these risk factors are measurable and largely modifiable, and thus continuing surveillance of the levels of risk factors is of fundamental importance in NCDs control [11].

In Afghanistan, due to years of conflict, it has been difficult to make any accurate estimates of NCDs prevalence including hypertension. According to recent studies conducted in Kabul for adults more than 40 years old and in Jalalabad for adults more than 25 years old, the prevalence of hypertension was 46.2% and 28.4%, respectively [12, 13]. Furthermore, Iran, being a neighbor of Afghanistan and a middle-income country, has estimated the prevalence of hypertension to be 22.1% [14]. Pakistan, another neighboring country, recorded the prevalence of high blood pressure as 25.3% [15]. There is no reason to believe that Afghans are less susceptible to NCDs than people in Pakistan and Iran. Hence, further research in this area will be very beneficial to health authorities. The purpose of this study is to estimate the prevalence of hypertension and assess the predictors in urban Afghan adults.

## 2. Methods and Materials

To provide estimates of hypertension and other factors of NCDs in Kabul city, a provincial cross-sectional study was conducted in November 2015 using the WHO STEP-wise approach revised instrument. This instrument prescribes three steps for assessing behavioral risk factors, physical measurements, and biological risk factors [16]. All permanent residents and household members aged more than 25 years, including men and women who gave consent to participate, were included in the study. Temporary residents (resident < 6 months), inhabitants of institutionalized settings, and insecure areas were excluded. Assuming the highest prevalence (50%), 95% confidence interval (CI), and margin of error of 5%, a sample size of 385 subjects was calculated to include in the study. However, considering the proportion of other risk factors and design effect (*D*_eff_ of 2) of cluster sampling, the final sample size was increased to (2 × 600) = 1200 for the city.

### 2.1. Sampling Strategy

The latest population census in Afghanistan was conducted in 1979 and hence is not useable now. Furthermore, the full and exact list of areas (villages) located in Kabul is not available. Consequently, it is difficult to estimate the overall population of residents in Kabul. However, a tentative estimate of the proportion of the study sample (*n* = 1202) among the general population of adult citizens was selected. The study team preferred to use the existing list the 2015 Expanded Programme for Immunization (EPI) of clusters as the sampling frame. This frame contains the estimated name of areas and number of households which is used for immunization. The strategy for approaching the 1200 households was determined to be cluster sampling of districts, clusters, areas, and households. At the first stage, from the list of 22 districts located in urban section of the city, the study team conventionally selected five districts using random numbers in the excel sheet. In the second stage from each selected district, the study team randomly selected the 2 clusters. Later on, the overall sample of 1200 households was divided among these selected areas according to the proportion to the size of household numbers in each areas. Finally, the households in each area were approached and selected systematically. Basically, therefore, the primary sampling unit was clusters of EPI, the secondary sampling unit was areas, and ultimate sampling unit was households. Lastly, within households, one adult individual with eligible criteria was chosen by the interviewer for enrollment in the study.

### 2.2. Data Collection

WHO STEP-wise tool contained questionnaires on demographic, behavioral, and physical measurements. Interviewers approached households, the final unit of sampling frame, for face to face data collection. A household was defined as a group of people who share the same food pot (not the same roof). In households with more than one eligible person, the study team used a lottery system to select only one respondent. In cases of refusal, the interviewer approached the next available household. Anthropometric measurements including height and weight were used to calculate body mass index (BMI). A BMI ≥ 30 kg/m^2^ was considered as obese, 25–30 kg/m^2^ as overweight, and 18.5–25 kg/m^2^ as normal weight [17]. A waist circumference of 94 cm for men and 80 cm for women was defined as central obesity [18]. Systolic blood pressure (SBP) 140 mmHg and diastolic pressure (DBP) 90 mmHg were considered as hypertensive. Furthermore, SBP of less than 120 mmHg and DBP of less than 80 mmHg were calculated as being hypotensive while the group between the two were considered as prehypertensive [19]. Blood samples were collected the next morning after the respondent had fasted for 10–12 hours and were transported in cold boxes (2–8°C) from field to Central Public Health Laboratory (CPHL) in Kabul. Using Cry-vials, the samples were coded with ID number of the questionnaire. On arrival in CPHL, all serum samples were stored at −80°C and later on were tested for triglyceride, cholesterol, and glucose. A fasting blood sugar of ≥126 mg/dL was considered as diabetes mellitus [20].* Epi*-*info*, version 7, and* SPSS*, version 20, were used for data management and analysis. The Institutional Review Board (IRB) reviewed the study protocol for technical and ethical approval and consent was taken from each individual before the interview. The results of physical and biochemical measurements communicated to required participants and the confidentiality of the information was maintained.

## 3. Results

The original total number of study participants was 1202 of which 30 records were taken from the study due to unavailability of sufficient epidemiological data or no blood; hence, analysis was conducted on 1172 records. Of these, 599 (51.1%) were females and 573 (48.9%) males with a mean age of 38.6 ± 12.2 years. Majority of the study participants were married (77.5%), while 60% of women were housewives and closely half of the respondents (49.6%) were illiterates (Table 1). Study subjects were reluctant to provide information on their income; however, it seems that just 7.6% had income of less than 10000 Afghani per month (150$).

Table 2 shows the prevalence of various behavioral risk factors for NCDs; 8.1% were current smokers and 46% of smokers had duration of 10 years. The general prevalence of snuff use was 9.8%; however, this figure is diluted due to low prevalence of snuff use in females (2%). Sixty-six percent had fruits and 65%% had vegetables less than three days per week. On average, the subjects were taking fruits 3.08 days per week and vegetables 3.10 days per week. Thirty-three percent of respondents reported to be using liquid oil for cooking in their kitchen while using solid ghee (52.1%) was more prevalent. Almost ten percent (9.4%) of the respondents practiced vigorous physical activity and 20.3% of subjects reported doing moderate physical activity. Approximately half (48%) of the respondents reported walking or using bicycle for 10 minutes per day. Forty-six percent of respondents (46.8%) recorded a reclining of three hours or more per day. About 57.5% of study respondents were overweight and obese and (60%) were suffering from central obesity. Only about 11.6% and 56.1% of the respondents had low or normal blood pressure, respectively, while 32.2% had high blood pressure. Out of this group of high blood pressure, 29.4% were unaware of their status of hypertension. Nine percent (9.1%) were recorded as raised blood sugar and of these the prevalence of diabetes was 9.3% for females and 8.9% for males. Approximately 30% had high level of cholesterol and 41% had high level of triglycerides. Furthermore, high level of low density lipoprotein (LDL) and high level of high density lipoprotein (HDL) were 42.2% and 48%, respectively, among study subjects.

Outside the descriptive analysis on this study, the bivariate analysis was computed for demographic and socioeconomic variables. As Table 3 shows on the bivariate analysis, age, sex, level of education, smoking, moderate physical activity, and taking red meat were found to be significantly associated with high blood pressure.

Furthermore, Table 4 shows that body mass index (BMI), central obesity, use of table salt, and high blood sugar (diabetes mellitus) were found to be significantly associated with hypertension at *P* value level of 0.05 or more.

In the multivariate logistic regression analysis, age, general obesity, central obesity, smoking, moderate physical activity, and taking fruits less than 3 days or more weekly were statistically significant predictors of hypertension.  Table 5 shows the adjusted odds ratio and 95% confidence interval along with *P* values.

## 4. Discussion

The main findings of this study were high level of hypertension among the adult population in Kabul city which is 32.2% despite being a low-income country. This finding is in line, and even higher, with other similar studies conducted elsewhere [13, 21, 22]. Mostly, attention is given on communicable diseases in the country while less focus is given to NCDs which impacts adversely on Afghanistan [23, 24]. In addition, the burden of hypertension in Kabul [12] in age group of 40 years and more was 46% which could be compared with same age group in this study. It appears that the high age groups are on greater risk of high blood pressure. About 29.4% of hypertension patients are unaware of their hypertension status indicating that more than one out of three cases of hypertension do not know that they have it. This finding is in agreement with other similar studies [25, 26].

Although many risk factors were significantly associated with hypertension in this study, at initial level however, few predictors were independently associated with hypertension. Age was independently associated with hypertension and the burden got high proportions as age increases. This is in line with other studies where the risks of hypertension increase with age [27, 28]. Furthermore, general and central obesity were statistically associated with hypertension and odds were high among respondents who were overweight and obese. This finding is in accordance and supported by other studies in similar settings [12, 26]. Smoking was also independently associated with hypertension. This finding is in agreement with other similar studies where substance use was a significant predictor of hypertension [29, 30]. Moreover, moderate physical activity and diet in terms of taking fruits were associated with hypertension as protective factors [31, 32].

This study identifies high prevalence of hypertension and some of the major factors associated with hypertension for people living in the urban areas of Kabul city. Most of these factors are modifiable as well as protective and these could be targeted to improve the health of the citizens. For instance, projects in relation to smoking, encouragement of physical activity, taking more fruits, and reducing obesity will be very useful in health improvement. Intervention inspiring education campaigns to raise awareness on physical activity and healthy diet as protective factors against all NCDs is recommended. Currently, due to urbanization, Kabul city is overcrowded with very limited jogging as well as aerobic sport centres. Establishment of such facilities, which are lacking in urban settings particularly for women, is encouraged.

Limitations to this study included financial constraints which prevented listing of the households ahead of study, overestimation of hypertension due to free checking of blood pressure and blood testing, and the poor security situation which forced the research team to exclude some areas.

## 5. Conclusion

As a whole, the findings of this study are very important to policy makers and planners in terms of prevalence and risk factors such as age, smoking, obesity, low physical activity, and dietary habits. Thus, effective community-based precautionary and control strategies will contribute to prevent hypertension.

## Figures and Tables

**Table 1:** Frequency distribution of demographic characteristics of the study participants, Kabul city (*N* = 1172).

Variables	Categories	Female		Male		Total	
		*N*	%	*N*	%	*N*	%
Age	25–34	301	50.3	291	50.8	592	50.5
	35–44	158	26.4	131	22.9	289	24.7
	45–54	89	14.9	76	13.3	165	14.1
	55+	51	8.5	75	13.1	126	10.8

Level of education	Illiterate	370	62.8	205	36	575	49.6
	Primary and unofficial	86	14.6	116	20.4	202	17.4
	Secondary school	72	12.2	154	27	226	19.5
	High school and over	43	7.3	75	13.2	118	10.2

Job categories	Official employees	53	8.9	120	21.1	173	14.8
	Students	7	1.2	10	1.8	17	1.5
	Private business	5	0.8	50	8.8	55	4.7
	Worker/farmer	1	0.2	76	13.3	77	6.6
	Jobless	9	1.5	52	9.1	61	5.2
	Housework	356	59.7	12	2.1	368	31.6
	Unable to work	165	27.7	250	43.9	415	35.6

Monthly income in AFN	Less than 10000	5	3.8	11	13.9	16	7.6
	More than 10000	127	96.2	68	86.1	195	92.4

Marital status	Single	54	9.1	82	14.5	136	11.7
	Married	460	77.7	437	77.2	897	77.5
	Widow/widower	43	7.3	11	1.9	54	4.7
	Divorced	4	0.7	2	0.4	6	0.5

**Table 2:** Frequency distribution of behavioral risk factors for noncommunicable diseases among the study participants, Kabul city (*N* = 1172).

Variables	Categories	Female		Male		Total	
		*N*	%	*N*	%	*N*	%
Cigarette smoking status	No	595	99.5	480	83.9	1075	91.9
	Yes	3	0.5	92	16.1	95	8.1

Duration of smoking in years	<10 years	7	100	56	50.5	63	53.4
	10–20 years	0	0	36	32.4	36	30.5
	≥20 years	0	0	19	17.1	19	16.1

Mouth snuff status	No	585	98	467	82.1	1052	90.2
	Yes	12	2	102	17.9	114	9.8

Fruit taking^1^ (days per week)	<3	372	64.7	378	68.1	750	66.4
	≥3	203	35.3	177	31.9	380	33.6

Vegetables taking^2^ (days per week)	<3	406	68.8	350	61.6	756	65.3
	≥3	184	31.2	218	38.4	402	34.7

Type of kitchen oil	Liquid	174	29.2	214	37.7	388	33.3
	Solid	326	54.7	281	49.5	607	52.1
	Both	95	12.3	70	12.3	165	14.2

Vigorous physical activity^3^	No	550	92.1	507	88.9	1057	90.6
	Yes	47	7.9	63	11.1	110	9.4

Moderate physical activity^4^	No	449	75.1	481	84.5	930	79.7
	Yes	149	24.9	88	15.5	237	20.3

Pedal or bicycle for 10 minutes per day	No	403	67.3	206	36	609	52
	Yes	196	32.7	366	64	562	48

Reclining/siting (hours per day)	<3	296	52.1	301	54.3	597	53.2
	≥3	272	47.9	253	45.7	525	46.8

^1,2^One serving is amount of fruits or vegetables taken once, ^3^physical activity in ten minutes caused high heart beats or respiration, and ^4^physical activity in ten minutes caused moderate heart beats or respiration.

**Table 3:** Bivariate analysis of biodemographic and socioeconomic factors and hypertension among study participants in Kabul, Afghanistan.

Variables	Categories	Normotensive	Hypertensive	Odds ratio	CI 95% LL	CI 95% UL
Age in years	25–34	495 (83.6)	97 (16.4)	1	Reference	
	35–44	170 (58.8)	119 (41.2)	3.572	2.595	4.918
	45–54	85 (51.5)	80 (48.5)	4.803	3.301	6.988
	55 and over	43 (34.1)	83 (65.9)	9.85	6.422	15.108

Gender	Female	379 (63.3)	220 (36.7)	1	Reference	
	Male	414 (72.3)	159 (27.7)	0.662	0.517	0.847

Level of education	Illiterate	368 (64)	207 (36)	1	Reference	
	Literate	394 (72.2)	152 (27.8)	0.686	0.533	0.883

Monthly income (Afghanis)	≤150 USD	11 (68.8)	5 (31.2)	1	Reference	
	≥150 USD	141 (72.3)	54 (27.7)	0.843	0.28	2.538

Smoking	No	712 (66.2)	363 (33.8)	1	Reference	
	Yes	79 (83.2)	16 (16.8)	0.397	0.229	0.69

Snuffing	No	722 (68.6)	330 (31.4)	1	Reference	
	Yes	67 (58.8)	47 (41.2)	1.535	1.034	2.278

Strong physical activity	No	717 (67.8)	340 (32.2)	1	Reference	
	Yes	73 (66.4)	37 (33.6)	1.069	0.705	1.62

Moderate physical activity	No	642 (69)	288 (31)	1	Reference	
	Yes	147 (62)	90 (38)	1.365	1.014	1.837

Sedentary lifestyle in hours daily	<3 hours	405 (67.8)	192 (32.2)	1	Reference	
	≥3 hours	352 (67)	173 (33)	1.037	0.807	1.331

Fruits serving days per week	<3 days	523 (69.7)	277 (30.3)	1	Reference	
	≥3 days	244 (64.2)	136 (35.8)	1.284	0.989	1.668

Vegetables serving days per week	<3 days	512 (67.7)	244 (32.3)	1	Reference	
	≥3 days	272 (67.7)	130 (32.3)	1.003	0.774	1.299

Taking red meat in days per week	<2 days	121 (89)	15 (11)	1	Reference	
	≥2 days	589 (65.7)	308 (34.3)	4.218	2.424	7.34

**Table 4:** Bivariate analysis of pathophysiologic factors and hypertension among study participants in Kabul city, Afghanistan.

Variables	Categories	Normotensive	Hypertensive	Odds ratio	CI 95% LL	CI 95% UL
Basic mass index	Underweight	26 (78.8)	7 (21.2)	1	Reference	
	Normal weight	360 (77.4)	105 (22.6)	1.083	0.457	2.566
	Overweight	285 (66)	147 (34)	1.916	0.812	4.518
	Obese	122 (50.4)	120 (49.6)	3.653	1.528	8.736

Central obesity (pregnant excluded)	No	367 (78.3)	102 (21.7)	1	Reference	
	Yes	426 (60.6)	277 (39.4)	2.34	1.792	3.054

Use of table salt	No	541 (65.8)	281 (34.2)	1	Reference	
	Yes	192 (72.7)	72 (27.3)	0.722	0.531	0.981

Diabetes mellitus	No diabetic	739 (69.4)	326 (30.6)	1	Reference	
	Diabetic	54 (50.5)	53 (49.5)	2.225	1.49	3.322

Total cholesterol	<190 mg/dL	559 (68.4)	258 (31.6)	1	Reference	
	≥190 mg/dL	234 (65.9)	121 (34.1)	1.12	0.86	1.459

Low density lipoprotein (LDL)	<100 mg/dL	458 (67.6)	220 (32.4)	0.953	0.746	1.218
	≥100 mg/dL	335 (67.8)	159 (32.2)	0.988	0.771	1.266

High density lipoprotein (HDL) borderline 40 mg/dL for male and 50 mg/dL for female	<40 and 50 mg/dL	409 (67.2)	200 (32.8)	1	Reference	
	≥40 and 50 mg/dL	384 (68.2)	179 (32.3)	0.953	0.746	1.218

Triglycerides	<150 mg/dL	476 (68.9)	215 (31.1)	1	Reference	
	≥150 mg/dL	317 (65.9)	164 (34.1)	1.145	0.894	1.468

**Table 5:** Multivariable analysis of risk factors and hypertension among study participants in Kabul city, Afghanistan.

Variables	Categories	Adjusted Odds ratio	CI 95% LL	CI 95% UL	*P* value
Age	Lower age	1	Reference		
	Higher age	1.081	1.067	1.096	<001

General obesity	No	1	Reference		
	Yes	0.474	0.328	0.685	<001

Central obesity	No	1	Reference		
	Yes	0.531	0.377	0.747	<001

Oil for kitchen	Liquid		Reference		
	Solid	1.698	1.234	2.338	<001

Smoking	No	1	Reference		
	Yes	2.232	1.152	4.326	<05

Moderate physical activity	No	1	Reference		
	Yes	0.671	0.461	0.977	<05

Fruits days per week	Less		Reference		
	High	1.098	1.014	1.188	<05
